# Editorial: Recent advances in the pathogenesis and potential biomarkers of fibrosis

**DOI:** 10.3389/fphar.2024.1527123

**Published:** 2024-12-04

**Authors:** Samar A. Antar, Aymen Halouani, Karim Tawfic, Walied Abdo, Ayman M. Mahmoud, Yassine Sassi

**Affiliations:** ^1^ Fralin Biomedical Research Institute at Virginia Tech Carilion, Roanoke, VA, United States; ^2^ Department of Pharmacy Practice, Faculty of Pharmacy, Horus University, New Damietta, Egypt; ^3^ Department of Pathology, Faculty of Veterinary Medicine, Kafrelsheikh University, Kafrelsheikh, Egypt; ^4^ Department of Life Sciences, Faculty of Science and Engineering, Manchester Metropolitan University, Manchester, United Kingdom; ^5^ Department of Biomedical Sciences and Pathobiology, Virginia-Maryland College of Veterinary Medicine, Virginia Tech, Blacksburg, VA, United States; ^6^ Department of Internal Medicine, VTC School of Medicine, Roanoke, VA, United States

**Keywords:** fibrosis, inflammation, collagen, extracellular matrix, matrisome

Fibrosis is the replacement of functional tissue architecture with excess fibrous connective tissue, leading to a reduction in organ function and ultimately organ failure and death (Antar et al., 2023). It originates when the extracellular matrix (ECM) of injured tissues has an excessive accumulation of fibrous connective tissue. Collagens, particularly types I and III, are the primary constituents of the fibrotic scar tissue. The fibrotic process is associated with chronic inflammation, metabolic homeostasis, and transforming growth factor-β1 (TGF-β1) signaling. Fibrosis is a pathological feature of most chronic inflammatory disorders and proinflammatory cytokines have been shown to be important initiators of fibrosis (Wynn and Ramalingam, 2012). TGF-β1 is a major driver of fibrosis through its actions on various cell types and through various signaling pathways (Meng et al., 2016). Prolonged healing in extremely fibrotic tissue results in tissue/organ dysfunction. Fibrotic tissue remodeling, associated with high morbidity and mortality, is often the source of organ malfunction. Preclinical models and clinical research in many organ systems have shown that fibrosis, which was long thought to be an irreversible and continuously developing process, is actually a very dynamic process (Henderson et al., 2020). A better understanding of the pathogenesis of fibrosis, the identification of noninvasive biomarkers, and the development of new therapeutic interventions are the current challenges in fibrosis research.

Ongoing research aims to better understand the molecular mechanisms of fibrosis, improve early detection, and develop targeted therapies to stop or reverse the fibrotic process. Fibrosis is a key feature in many chronic diseases and is often associated with poor prognosis due to its irreversible nature and the critical role it plays in tissue and organ dysfunction. Experimental models are crucial for understanding the pathophysiological mechanisms of fibrosis and for exploring potential therapeutic targets. Model organisms, especially mice, provide an invaluable resource for both basic and applied research, enabling studies on disease prediction, modeling, and the identification of underlying mechanisms. Since drug development necessitates testing on these models before progressing to clinical trials, animal models that recapitulate the features of the human disease are essential for advancing the field. Similarly, the development of new techniques and methods to study the pathways involved in the fibrotic process will contribute to better understand the pathogenesis of fibrotic diseases.

This Research Topic encompasses three research articles and two review articles. These articles introduced cutting-edge research on fibrosis. The guest editors are pleased to present a compendium of these articles as follows;

In this special Research Topic of Frontiers in Pharmacology, Wei et al. discussed the role of Transient receptor potential ankyrin1 (TRPA1) in fibroblasts and described its relationship with fibroblast activation, with a particular focus on the interactions between TRPA1 and inflammatory factors. The authors reviewed the modulation of fibrosis by TRPA1 in multiple diseases including cardiac, synovial, pulmonary and other fibrotic diseases. The authors highlighted that TRPA1 has different functions in the fibrosis process across different organs and provided intriguing insights into the mechanisms of TRPA1 in fibroblasts. Additionally, they discussed how TRPA1 could serve as a potential target for treating fibrosis and reviewed the agonists and antagonists of TRPA1.

Elmorsy et al. explored the potential therapeutic effects of itraconazole (ITRCZ)-loaded nanoparticles in reducing liver fibrosis by modulating the Hedgehog pathway. ITRCZ has many limitations, such as poor solubility and bioavailability. The authors formulated and optimized chitosan nanoparticles (Cht NPs) loaded with ITRCZ to increase the efficacy of ITRCZ for treating liver fibrosis. The optimized formulation increased primary hepatocyte survival *in vitro*, and improved liver function in a rat model induced by thioacetamide (TAcA) by decreasing fibrosis and attenuating the hepatic expression of Hedgehog signaling components. These findings raise the possibility of using ITRCZ-Cht NPs as a new antifibrotic treatment for liver fibrosis.

Ávila-Martínez et al. summarized the crucial role of the renin-angiotensin system (RAS) in resolving inflammatory and fibrotic diseases, offering insights into its different components as potential therapeutic options. The authors described the impact of different components of the counter-regulatory axis of the RAS on different pathologies. Moreover, the authors addressed the translational significance of using peptides of the renin-angiotensin system as anti-inflammatory and anti-fibrotic treatments and noted that studies addressing the role of peptides of the counterregulatory axis of the renin-angiotensin system in the control of inflammatory and fibrotic diseases remain limited. Finally, the authors described the challenges that remain before the use of peptides of the counterregulatory axis of the renin-angiotensin system as anti-inflammatory and anti-fibrotic treatments to stop or reduce the progression of diseases.

Ma et al. investigated the effects of the anti-inflammatory and anti-fibrotic drug cryptotanshinone (CTS) on inflammation and fibrosis in bronchopulmonary dysplasia (BPD). BPD is a chronic lung disease affecting premature infants, and is pathologically characterized by inflammation and fibrosis. CTS reduced hyperoxia-induced pulmonary fibrosis and inflammation in Sprague–Dawley neonatal rats by regulating the expression of pro-inflammatory and pro-fibrotic factors in macrophages. The authors concluded by highlighting the therapeutic potential of CTS in the treatment of BPD.

By employing a new two-step workflow, Zhang et al. identified key matrisome components that affect liver stiffness. The authors used a new approach that combines their previously enhanced sodium dodecyl sulfate (ESDS) decellularization technique with the conventional SDS method. Using this method, they identified midly and highly insoluble matrisome members, including collagens, glycoproteins, proteoglycans and ECM regulators and associated proteins in the mouse liver. Subsequent regulatory network and functional enrichment analyses identified the pathways associated with highly insoluble matrisome members. This promising approach should be employed in future studies designed to identify midly and highly insoluble matrisome members and their associated pathways in liver (and other organs) fibrosis.

The editors expect the articles published in this Research Topic to capture the interest of readers and believe that researchers will gain valuable insights, advancing their understanding of the mechanisms underlying fibrosis and fostering the development of new therapeutic approaches.
